# Headache disorders are third cause of disability worldwide

**DOI:** 10.1186/s10194-015-0544-2

**Published:** 2015-06-25

**Authors:** Timothy J. Steiner, Gretchen L. Birbeck, Rigmor H. Jensen, Zaza Katsarava, Lars J. Stovner, Paolo Martelletti

**Affiliations:** Department of Neuroscience, Norwegian University of Science and Technology, Edvard Griegs Gate, Trondheim, Norway; Division of Brain Sciences, Imperial College London, London, UK; Chikankata Hospital, Mazabuka, Zambia; Department of Neurology, University of Rochester, Rochester, NY USA; Department of Neurology, University of Copenhagen, Glostrup Hospital, Glostrup, Denmark; Department of Neurology, University of Duisburg-Essen, Essen, Germany; Department of Neurology, Evangelisches Krankenhaus, Unna, Germany; Norwegian Advisory Unit on Headache, Department of Neurology and Clinical Neurophysiology, St Olavs University Hospital, Trondheim, Norway; Department of Clinical and Molecular Medicine, Sapienza University, Rome, Italy; Regional Referral Headache Centre, Sant’Andrea University Hospital, Rome, Italy

From time to time, there is news that is of particular consequence to all people affected by headache.

In December 2012, *Lancet* published the Global Burden of Disease Study 2010 (GBD2010). We wrote then [1]: “Few reports are likely to have more profound meaning for people with headache, or carry greater promise for a better future, than the seven papers (and one in particular [2]) that were presented.” So it was: the essential finding – that migraine was the seventh highest specific cause of disability worldwide – has been widely cited in both scientific and informal literature, pointedly noted by health commentators, trumpeted loudly by lay organisations and quietly harnessed by those seeking grants for headache research. It has given legitimacy to arguments that headache disorders contribute in a big way to public ill-health and disability [3], and strong backing to pleas for political recognition of this fact [4].

Now there is more, from the Global Burden of Disease Study 2013 (GBD2013), and it is of similarly arresting significance. Published in *Lancet* earlier this month [5], its key findings for those whose interests focus on headache are threefold: migraine is the *sixth* highest cause of disability worldwide; medication-overuse headache (MOH) is included in these surveys for the first time and enters the top twenty causes of disability at 18^th^; and adding together just these two puts headache disorders *third* among the worldwide causes of disability, measured in years of life lost to disability (YLDs).

Thus in the 23 years of the Global Burden of Disease project (GBD), from 1990 to 2013, headache has come from nowhere – wholly ignored, not thought even worth measuring – into the leading three of the several hundred contributors to the global burden of disease that GBD counts. The background and a little of the history of this extraordinary transition should be told. What we are reporting here are the outcomes of huge, sustained, coordinated effort.

GBD itself is a massive, ongoing, iterative enterprise [6]. It was undertaken initially, in 1990 and 2000, by the World Health Organization (WHO) but now is led by the Institute of Health Metrics and Evaluation (IHME) of the University of Washington, Seattle, WA, USA. Its stated purpose now is to set out “a comprehensive picture of what disables and kills people across countries, time, age, and sex”; towards this, it provides “a tool to quantify health loss from hundreds of diseases, injuries, and risk factors, so that health systems can be improved and disparities can be eliminated” [6]. The data in GBD2013 came through a consortium of more than 1,000 researchers in over 100 countries, capturing premature death and disability in 188 countries from more than 300 diseases [6]. Our objective has been to secure among these diseases the rightful inclusion of migraine, tension-type headache (TTH) and MOH: headache disorders that we know cause substantial disability [3].

The Global Campaign against Headache was launched in 2003 with a clear ultimate purpose: to reduce the burden of headache worldwide [7]. At that time, 12 years ago, it was not at all clear what this burden was, either in scope or scale. As a result of some lobbying during discussions with WHO in the years prior to the Campaign’s launch, GBD2000 included migraine [8]. No other headache disorders made it, but this was nevertheless a major advance for those concerned about headache, not just because GBD1990 had ignored headache totally but far, far more because migraine was found – on the evidence submitted – to be in the leading 20 causes (19^th^) of disability worldwide [8]. This “discovery” propelled headache disorders into WHO’s priorities [3, 4].

For the Campaign this was merely a call to arms, because also clear at that time was that the evidence submitted to GBD2000 was seriously deficient. Of course it related only to migraine, which was neither the most prevalent nor the most disabling of headache disorders, but this was not the issue. Migraine was certainly the best studied of the headache disorders, from all aspects including epidemiologically, and the epidemiological evidence then available from all studies of acceptable quality had been thoroughly collated (it was later published as a review [9]). The problem was that it focused strongly on North and South America and Western Europe, with a small Far-East cluster of studies in Japan, Taiwan and the Korean peninsula; left unrepresented were most of the Western Pacific Region (including mainland China), all of South East Asia (including India), all of the Eastern Mediterranean Region, most of Africa and all of Eastern Europe (including Russia). The people unrepresented in these territories were more than half the world’s population.

Not long after the Global Campaign launched, data collection began for GBD2010 (which was initially to be GBD2005). Filling the largest of the data gaps was therefore the first priority of *Lifting The Burden* (LTB), the UK-registered charity conducting the Campaign [10, 11]. LTB had two objectives for GBD2010: to secure inclusion of the other headache disorders of public-health importance – TTH and MOH – and to show, as we then believed, that headache disorders collectively were among the top ten causes of disability worldwide. It became something of a race against time, firstly to develop the methodology for population-based door-to-door studies with a validated diagnostic questionnaire based on ICHD-II [12] and then to implement it in the big countries: China, India and Russia, home to 2.5 billion people. These things were done, and, with much better information, GBD2010 reported migraine more realistically as the seventh highest specific cause of disability measured in YLDs [1, 2]. This of course achieved LTB’s first objective. As for the second, TTH was included in the survey, but with a very low disability weight (DW) allocated to it. GBD2010 reported TTH as the second most prevalent disorder in the world (after dental caries), and migraine third [2], but despite this TTH added rather little compared with migraine to the global disability burden. What about MOH? This was initially included but not in the end reported because, it was argued – correctly, we believe, that prevalence data were not good enough to support regional estimates of burden attributable to this disorder. The particular difficulties of estimating MOH prevalence were recently discussed [13, 14]. Nonetheless, a DW was allocated to MOH, which was of crucial importance when it came to GBD2013.

In the interim, between GBD2010 and GBD2013, LTB had supported further Global Campaign studies in Nepal in South East Asia, in Saudi Arabia and Pakistan in Eastern Mediterranean and in Zambia and Ethiopia in Africa – huge knowledge gaps – while collaborating with GBD in collating data published by other workers. The particular importance of the LTB studies lay in their use of similar methodology [15], the inclusion of MOH in their enquiries and the purposive selection of countries for survey. GBD2013 was therefore considerably better informed than GBD2010, not only with more comprehensive regional data but also, and in particular, with greatly enhanced data on MOH (and a DW available from GBD2010 for YLD estimates).

LTB has prioritised this work on data gathering and our collaboration with GBD above all else. It has involved multiple complex studies in all world regions, and taken most of our resources, but as a policy we believe it has been strategically correct. If the ultimate purpose of the Campaign is to reduce the burden of headache worldwide, it must first be known what this burden is – the Campaign’s first objective [7]. At the same time, working with GBD does much to achieve the Campaign’s second objective, which is creation of awareness of this burden. Indeed this work of data gathering continues, with studies ongoing or planned in countries in Central and South America, North and West Africa and South East Asia. We have not forgotten children and adolescents, for which studies can be school-based [16]. All of these will not only benefit future iterations of GBD but also, just as importantly, serve as needs-assessment studies informing health policy locally, in the countries and regions where the data are gathered.

To end, it would be easy to claim the findings of GBD2013 as a triumphal conclusion of prolonged hard effort, since on a technical level they are, but that would overlook their tragic meaning. As we reported earlier [1], GBD measures disease burden as it is – alleviated by whatever treatments are made available. Headache disorders, we said at the time of GBD2010, were among the top ten causes of disability because they were common and disabling, but we asked: “For what conceivable reason do headache disorders remain among these ignominious top ten when they are largely treatable?” [1].

Now we must ask the chastening question: “Why are they among the top three?”
